# EDG2 enhanced the progression of hepatocellular carcinoma by LPA/PI3K/AKT/ mTOR signaling

**DOI:** 10.18632/oncotarget.19825

**Published:** 2017-08-02

**Authors:** Meng Xu, Zhikui Liu, Cong Wang, Bowen Yao, Xin Zheng

**Affiliations:** ^1^ Department of Hepatobiliary Surgery, The First Affiliated Hospital of Xi’an Jiaotong University, Xi’an, Shaanxi 710061, China

**Keywords:** EDG2, HCC, post-surgical prognosis, PI3K/AKT/mTOR, EMT

## Abstract

HCC is the leading type of the malignant liver tumors with the unsatisfied prognosis. Liver resection has been considered as the predominant curative therapy, however, the post-surgical prognostic evaluation remains an urgent problem and the mechanism of HCC metastases has not been understood completely. EDG2 has been found to accelerate tumor progression through mediating different cell pathways, however, it remains unclear about the role of EDG2 on hepatocarcinogenesis. Here, EDG2 expression was found increased notably in HCC tissues by immunohistochemistry compared with adjacent liver tissues and comparison of survival curves revealed that EDG2 upregulation in HCC tissues was associated with the worse prognosis after liver resection. The positive correlation between EDG2 up-regulation and EMT was observed in HCC samples. Furthermore, EDG2 over-expression in HCC cells brought the typical EMT characteristics including up-regulation of Vimentin, Fibronectin and N-cadherin, suppression of E-cadherin, and enhanced cell migration and invasion capacities. Knockdown of EDG2 reversed the EMT phenotype in HCC cells. The *in vivo* experiments also identified the oncogenic role of EDG2 on HCC growth. The mechanistic studies elucidated that EDG2 enhanced mTOR phosphorylation via PI3K/AKT signaling and consequently induced EMT of HCC cells. Moreover, EDG2 was found to promote cell viability and proliferation of HCC cell through PI3K/AKT/mTOR/Skp2/p27^Kip1^ signaling. Taken together, the data here demonstrated EDG2 was a potential predictor for HCC patients receiving liver resection and accelerated HCC progression via regulating EMT driven by PI3K/AKT/mTOR signaling.

## INTRODUCTION

Hepatocellular carcinoma (HCC) has been identified as the sixth leading cancer and the second most common cause of cancer-related mortality due to its unfavorable prognosis [1]. During this decade, its incidence remains increasing in several countries, particularly in China which accounts for more than half of the total number of cases and deaths [2]. Although the surgical techniques and instruments for HCC surgery have been improved dramatically recently, the prognosis of HCC patients after liver resection remains unsatisfied, with an approximately 38–61% depending on the HCC stage [3]. Therefore, it is urgent to identify the biomarkers predicting HCC survival after liver resection and decipher their underlying molecular pathways contributing to HCC progression in order to establish the rational targeted therapeutic strategies following liver resection.

The concept of epithelial-mesenchymal transition (EMT) was raised by Elizabeth Hay in the early 1980s, which was described that epithelial cells losted epithelial markers and acquired mesenchymal characteristics [4]. A growing body of evidences have verified that EMT plays a critical role in accelerating metastasis in epithelium-derived carcinoma [5–7]. Our previous investigation revealed that EMT induced by GLI1/SNAI1 signaling promoted recurrence and metastases of HCC after surgery [8]. Although EMT has been considered as a critical event during HCC metastases by several studies, the induction mechanisms of EMT in HCC are still poorly understood.

Endothelial differentiation gene 2 (EDG2), also known as LPA1, is the first established as a kind of G protein-coupled receptor for lysophosphatidic acid (LPA) which was isolated from a mouse cerebral cortical neuroblast in 1996 [9]. Then, several groups found positive EDG2 expression in a variety of adult tissues [10], including liver [11]. Recent studies identified EDG2 as an oncogenic factor in several cancers. It has been found that EDG2 over-expression caused a high frequency of late-onset, estrogen receptor (ER)-positive on the model of transgenic mice and increased the invasion and metastasis capacities of tumor cells [12]. Moreover, another mechanistic investigation revealed that EDG2 promoted the metastases of breast cancer via activating ZEB1/miR-21 signaling [13]. The studies about ovarian cancer showed that EDG2 mediated cell proliferation, migration, or invasion of tumor cells positively through up-regulating FOXM1 [14]. Knockdown of EDG2 was found to decrease the incidence of pulmonary melanoma metastases [15]. In gallbladder cancer, EDG2 was demonstrated to be over-expressed in the muscle invasive bladder cancer specimen compared with the non-muscle invasive specimens and enhanced invasion ability of tumor cells by up-regulating ROCK1 expression and myosin light chain phosphorylation [16].

During liver resection, platelets accumulated in remnant liver tissues and secreted numerous LPA which has been confirmed to contribute to EMT induction in ovarian carcinoma [17, 18]. It seemed that LPA/EDG2 axis might be able to drive EMT in remnant HCC tissues and implicated with HCC recurrence and metastases after liver resection. Therefore, in the present investigation, we were sought to identify the expression of EDG2 in HCC tissues and its relationship with HCC prognosis, and explore the relevant underlying molecular mechanisms.

## RESULTS

### EDG2 was up-regulated magnificently in HCC cell lines and tissues

**Figure 1:**
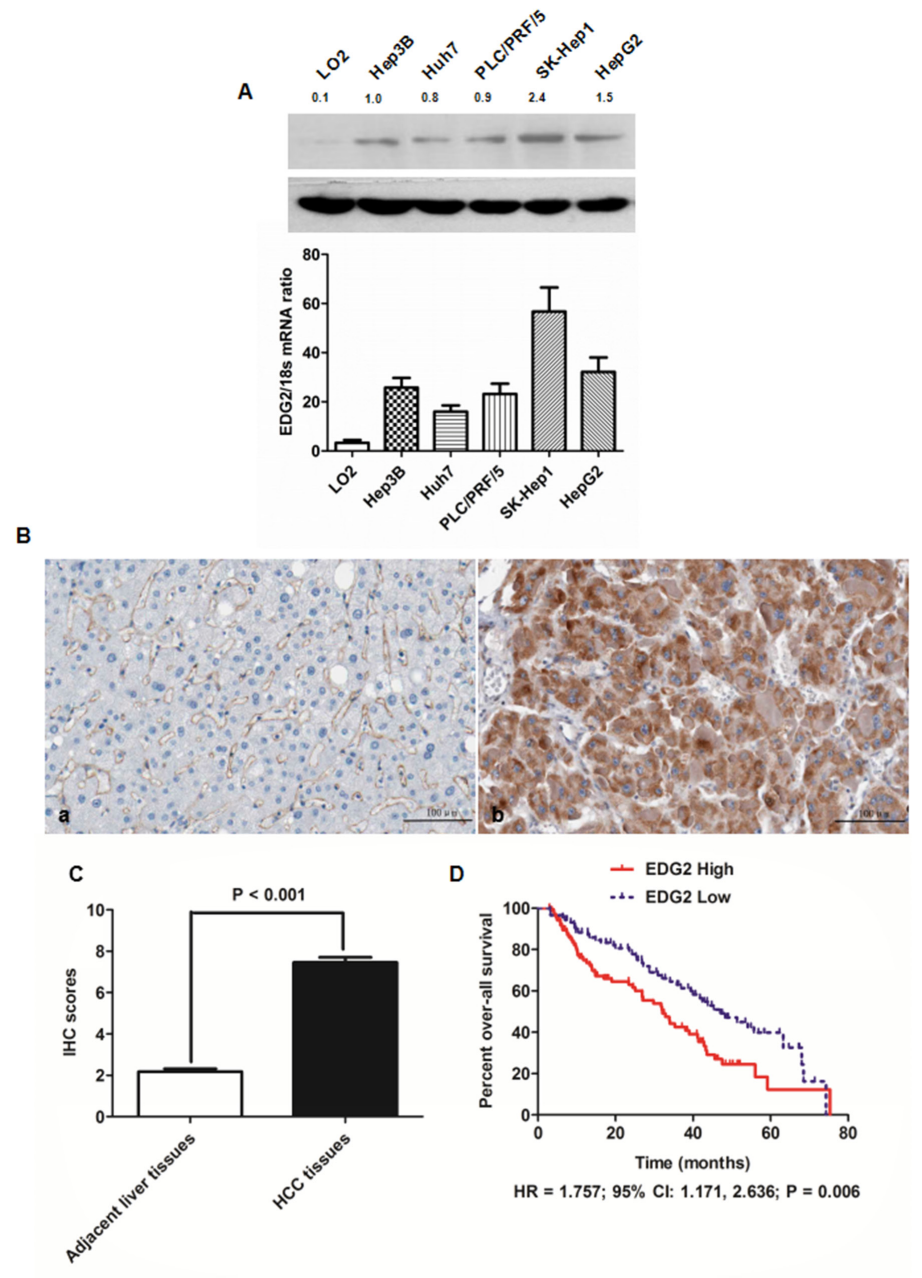
EDG2 was over-expressed in both HCC cell lines and HCC tissues **(A)** As assessed by both Western immunoblotting and qRT-PCR, there was significantly more EDG2 expression in all HCC cell lines (including Hep3B, Huh7, PLC/PRF/5, SK-Hep1 and HepG2) in contrast to normal liver cell LO2 at the level of protein and mRNA. **(B)** The left panel was the representative picture of EDG2 IHC staining in adjacent liver tissues, while the representative picture of EDG2 IHC staining was the right panel. It was notably that EDG2 expression was up-regulated aberrantly in HCC tissues compared to adjacent liver tissues. **(C)** Mann-Whitney U test displayed that there was remarkably more EDG2 expression in HCC tissues than adjacent liver tissues. **(D)** Comparison of Kaplan-Meier survival curves demonstrated that HCC patients from EDG2 high group suffered from the worse over-all survival after liver resection than those from EDG2 low group (HR = 1.757; 95% CI: 1.171, 2.636; P = 0.006).

To investigate the expression of EDG2 in HCC cells and human liver cells, both qRT-PCR and western immunoblotting were carried out. There was more mRNA and protein expression of EDG2 in 5 HCC cell lines than L02 cell, a sort of immortalized human normal liver cell. SK-Hep1 cells had the highest level of EDG2 expression among the 5 HCC cells, therefore, SK-Hep1 cell line was used as the cell model in the EDG2 knockdown experiment here (Figure 1A). On the other hand, there was the related lower level of EDG2 expression in both Huh7 and Hep3B cells. Hence, both Huh7 and Hep3B cells were used in EDG2 enforced-expression assays.

Next, we detected the EDG2 expression with IHC staining in HCC tissues (n = 210) as well as in corresponding adjacent liver tissues. Strong EDG2 expression was detectable in cytoplasm of tumor cells, with normal liver cells in adjacent liver tissues being weak positive or negative for EDG2 expression (Figure 1B). After analyzing the IHC scores by Mann-Whitney U test, we found that EDG2 expression in HCC tissues was markedly higher than adjacent liver tissues (Figure 1C), which was consistent with the results of the examination about EDG2 expression on HCC cell lines.

### Higher EDG2 expression in HCC tissues was associated with the poor clinical characteristics and prognosis of HCC patients

To further examine whether EDG2 was a potential post-surgical predictive factor for HCC patients, we estimated the correlation between EDG2 expression in HCC tissue and clinicopathological features of HCC patients. As shown in Table 1, we divided 210 HCC patients into two groups: high EDG2 group in which patients expressed more EDG2 expression in HCC tissues than adjacent liver tissues, and low EDG2 group in which there was negative or less EDG2 expression in HCC tissues compared with adjacent liver tissues. It was shown that there were more patients in high EDG2 group with high level of serum AFP (84.5% *vs.* 62.1%, P = 0.004), larger tumor diameter (69.1% *vs.* 31.0%, P < 0.001), high Edmonson-Steiner classification (69.1% *vs.* 20.7%, P < 0.001), advanced TNM stage (78.5% *vs.* 24.1%, P < 0.001), portal vein invasion (51.9% *vs.* 13.8%, P = 0.021), and intra-hepatic metastases (23.2% *vs.* 3.4%, P = 0.014). The follow-up information was obtained from 175 of 210 HCCs and the median follow-up time was 42.5 months, ranging from 3 to 75.4 months. We classified the 175 HCC patients into two groups: EDG2 high group and EDG2 low group using the median IHC score of EDG2 in HCC tissues as the cut-off value. The 1-, 3-, and 5-year survival rates of patients from EDG2 high group were less than those of EDG2 low group (60% *versus* 72%, 27% *versus* 42%, and 3% versus 12%, respectively). Comparison of Kaplan-Meier survival curves also demonstrated that HCC patients from EDG2 high group suffered from the worse over-all survival after liver resection than those from EDG2 low group (HR = 1.757; 95% CI: 1.171, 2.636; P = 0.006; Figure 1D).

**Table 1:** Demographic information and clinical characteristics of 210 HCC patients

Clinicopathological features		No. of patients		χ^2^	P
		High EDG2	Low EDG2		
Age (years)	< 50	76	10	0.582	0.445
	≥ 50	105	19		
Gender	Male	124	17	1.108	0.293
	Female	57	12		
HBV infection	Present	129	23	0.808	0.369
	Absent	52	6		
Serum AFP level (ng/mL)	< 400	28	11	8.339	0.004
	≥ 400	153	18		
Tumor diameter (cm)	< 5	56	20	15.65	< 0.001
	≥ 5	125	9		
Liver cirrhosis	Present	150	21	1.808	0.179
	Absent	31	8		
Edmondson-Steiner classification	I + II	56	23	24.921	< 0.001
	III + IV	125	6		
TNM stage	I + II	39	22	35.781	< 0.001
	III + IV	142	7		
Portal vein invasion	Present	94	4	5.309	0.021
	Absent	117	25		
Intra-hepatic metastases	Present	42	1	5.991	0.014
	Absent	139	28		

A series of univariate Cox proportional hazards model analyses were performed and revealed that the worse prognostic factors included advanced TNM staging, portal vein invasion, intra-hepatic metastases, and higher EDG2 expression in tumor tissues. And multivariate analysis further identified portal vein invasion, intra-hepatic metastases, and higher EDG2 expression in tumor tissues as the independent prognostic factors for HCC patients after liver resection (Table 2). Taken together, these results suggested forcefully that EDG2 contributed into HCC progression dramatically and was a potential effective post-surgical predictor for HCC patients.

**Table 2:** Univariate and multivariate analyses of prognostic factors in HCC patients after liver resection

Clinicopathological features	Univariate analysis		Multivariate analysis	
	RR (95% CI)	P value	RR (95% CI)	P value
Age, year (<50 versus ≥50)	0.985 (0.523 - 1.698)	0.215	0.879 (0.497-1.538)	0.344
Gender (female versus male)	0.214 (0.085 – 1.052)	0.169	0.234 (0.099 – 1.106)	0.205
HBV infection	1.624 (0.897 – 1.689)	0.087	1.529 (0.745 – 1.429)	0.092
Serum AFP level (ng/mL) (≥ 400 versus < 400)	1.981 (0.987 - 2.681)	0.062	1.748 (0.878 – 1.540)	0.056
Tumor diameter (cm) (≥ 5 versus < 5)	2.158 (0.912 - 2.801)	0.051	1.908 (0.854 – 2.607)	0.077
Liver cirrhosis	1.533 (0.817 – 2.192)	0.084	1.301 (0.568-1.872)	0.095
Edmondson-Steiner classification (III + IV versus I + II)	1.687 (0.859 – 2.325)	0.107	2.157 (1.155 – 3.255)	0.115
Advanced TNM staging	1.632 (1.026 – 3.015)	0.042	1.354 (0.898 – 2.411)	0.081
Portal vein invasion	2.321 (1.523 – 3.544)	0.004	2.011 (1.341 – 3.017)	0.017
Intra-hepatic metastases	3.215 (2.218 – 4.028)	0.001	3.008 (2.012 – 3.752)	0.005
Higher EDG2 in tumor tissue	2.195 (1.485 – 3.658)	0.001	1.658 (1.035 – 2.958)	0.001

### EDG2 accelerated the cell viability, proliferation, migration and invasion of Huh7 cells through driving EMT phenotype

**Figure 2:**
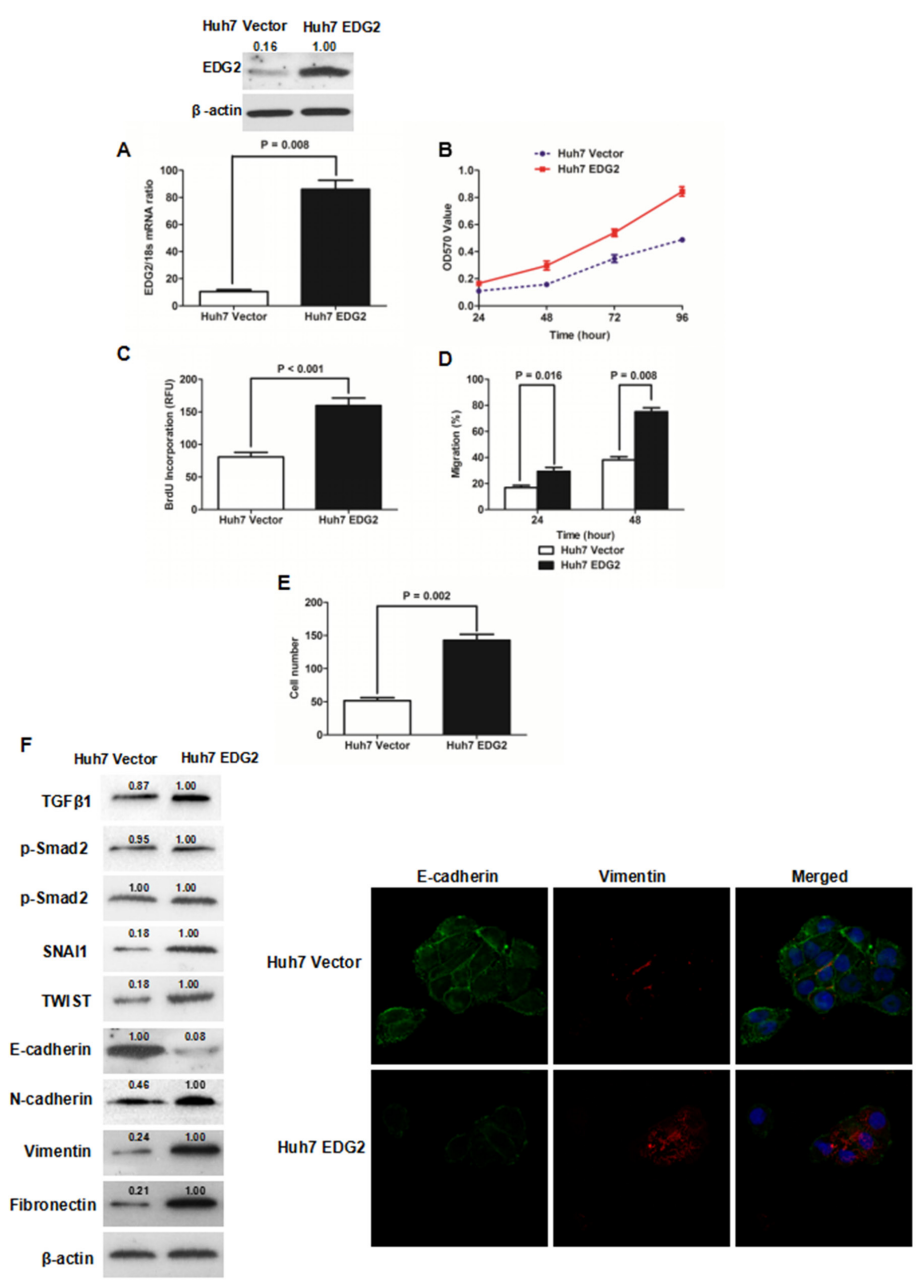
Enforced expression of EDG2 promoted cell growth and induced EMT phenotype of Huh7 cells **(A)** Transfection of EDG2 plasmidincreased EDG2 expression apparently in Huh7 cells. **(B)** MTT assay showed that over-expression of EDG2 enhanced the viability of Huh7 cells at 48, 72 and 96 hours notably. **(C)** ELISA assay found that BrdU incorporation of Huh7 cells was increased by EDG2 over-expression. **(D)** As assessed by wound healing assay, it was proven that migration of Huh7 cells was boosted by enforced expression of EDG2. **(E)** More Huh7 cells were found in the lower surface of the membrane after enforced expression of EDG2 in Transwell invasion assay. **(F)** Western immunoblotting assay demonstrated that there was less E-cadherin expression and more expression of N-cadherin, Vimentin and Fibronectin in Huh7 EDG2 cells than Huh7 Vector. And EDG2 overexpression up-regulated both SNAI1 and TWIST without activating TGFβ1 signalings. Meanwhile, immunofluorescent staining also comfirmed that forced expression of EDG2 in Huh7 cells leaded to both down-regulatioin of E-cadherin and up-regulation of Vimentin.

To address the function of EDG2 on the pathogenesis of HCC, we built Huh7 cell model with enhanced EDG2 expression via transfecting EDG2 expressing plasmid. As shown in Figure 2A, both qRT-PCR and western immunoblotting showed that there was significantly more EDG2 expression in Huh7 EDG2 cells than Huh7 Vector cells. As assessed by MTT assay, cell viability of Huh7 cells was increased by enforced expression of EDG2 apparently at 24, 48, 72 and 96h (Figure 2B). Consistently, EDG2 over-expression promoted cell proliferation of Huh7 cells greatly (P = 0.002, Figure 2C). The wound healing assay displayed that cell migration of Huh7 was enhanced by EDG2 up-regulation (Figure 2D, Supplementary Figure 1A). And enforced expression of EDG2 up-regulated invasion ability of Huh7 cells as examined by Transwell chamber assay with Matrigel (Figure 2E, Supplementary Figure 1B). These data indicated that EDG2 mediated positively HCC cell migration and invasion. Next, we investigated the effect of EDG2 on EMT phenotype which had been found attributed to HCC metastases [23]. Western immunoblotting assay demonstrated that there was less E-cadherin expression and more expression of N-cadherin, Fibronectin and Vimentin in Huh7 EDG2 cells than Huh7 Vector cells (Figure 2F). Although it seemed that enhanced expression of EDG2 did not influence TGFβ1 signalings, EMT transcription factors including SNAI1 and TWIST were both found up-regulated by overexpression of EDG2 in Huh7 cells. Immunofluorescent staining also confirmed that EDG2 over-expression reduced the expression of E-cadherin in Huh7 cells while increasing Vimentin expression (Figure 2F).

### Knockdown of EDG2 inhibited cell viability, proliferation, migration and invasion and revertedEMT phenotype in SK-Hep1 cells

**Figure 3:**
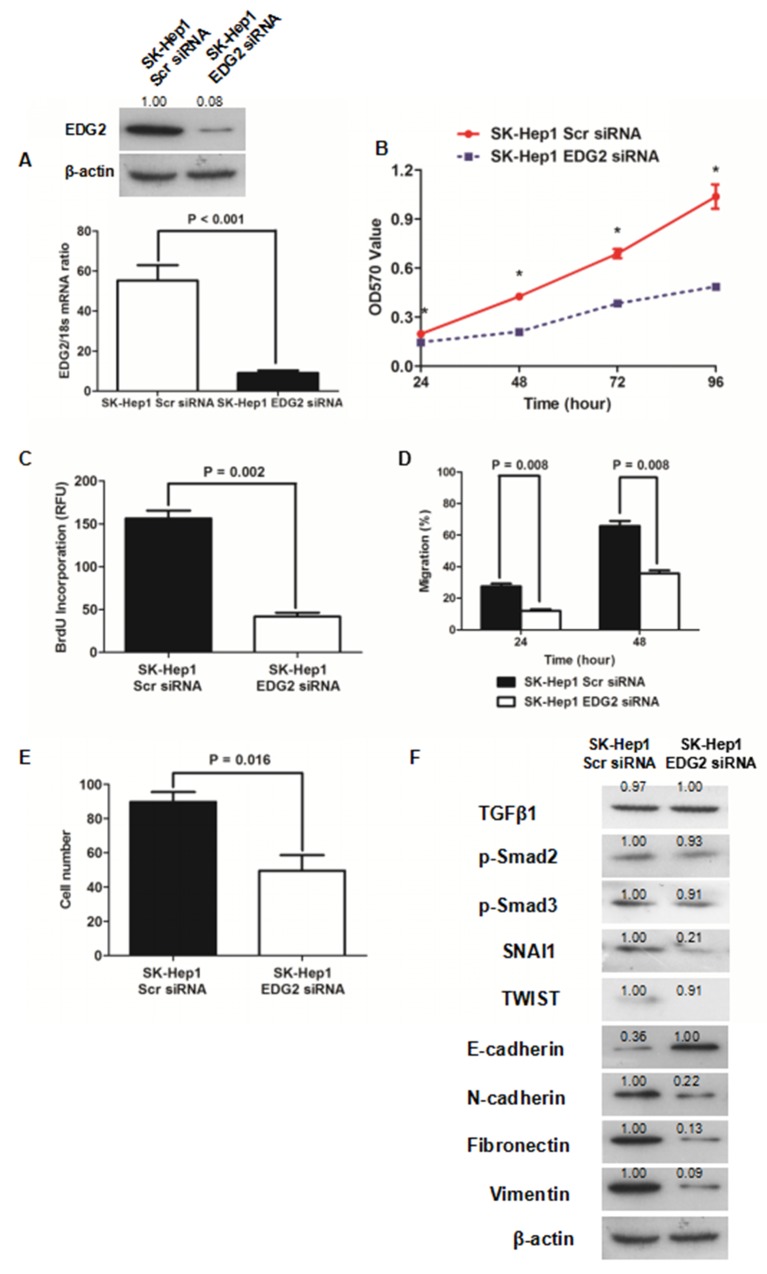
Knockdown of EDG2 repressed cell viability and proliferation and inversed EMT of SK-Hep1 **(A)** siRNA sequences suppressed EDG2 expression in SK-Hep1 cells markedly. **(B)** It was found cell viability of SK-Hep1 cells was inhibited dramatically by knockdown of EDG2 by MTT assay. **(C)** Knockdown of EDG2 was also found to inhibit cell proliferation of SK-Hep1 cells as examined by BrdU incorporation assay. **(D)** Wound healing assay demonstrated that migration capacity of SK-Hep1 cells was attenuated by silencing EDG2 significantly. **(E)** Transwell invasion assay showed that knockdown of EDG2 leaded to suppression of cell invasion ability of SK-Hep1 cells. **(F)** By Western immunoblotting assay, EDG2 silencing brought about more E-cadherin expression and less expression of N-cadherin, Fibronectin, and Vimentin of SK-Hep1 cells while inhibiting the expression of EMT transcription factor SNAI1 and TWIST. However, knockdown of EDG2 in SK-Hep1 cells did not repress the expression of TGFβ1, phosphorylated Smad2 and phosphorylated Smad3, which implied that EDG2 did not activate TGFβ1 signalings.

To further determine whether EDG2 induced EMT phenotype of HCC cells, we silenced the EDG2 expression by transfection of siRNA sequences (Figure 3A). MTT assay showed that knockdown of EDG2 reduced cell viability of SK-Hep1 cells significantly (Figure 3B). As examined by ELISA assay, BrdU incorporation of SK-Hep1 cells was repressed by transfecting siRNA sequences against EDG2 (Figure 3C), which indicated that EDG2 knockdown leaded to suppression of SK-Hep1 cell proliferation. EDG2 knockdown also suppressed the cell migration and invasion abilities of SK-Hep1 cells (Figure 3D and 3E, Supplementary Figure 2A and 2B). Western immunoblotting assay displayed that there was more E-cadherin expression and less expression of N-cadherin, Fibronectin and Vimentin in SK-Hep1 EDG2 siRNA cells than SK-Hep1 Scr siRNA cells (Figure 3F). And both TWIST and SNAI1 were repressed by knockdown of EDG2. Consistently, TGFβ1 signaling was also not found impacted by silencing EDG2 in SK-Hep1 cells. These data confirmed the oncogenic function of EDG2 on HCC progression by driving EMT phenotype.

### EDG2 was essential for LPA to induce EMT in HCC cells

**Figure 4:**
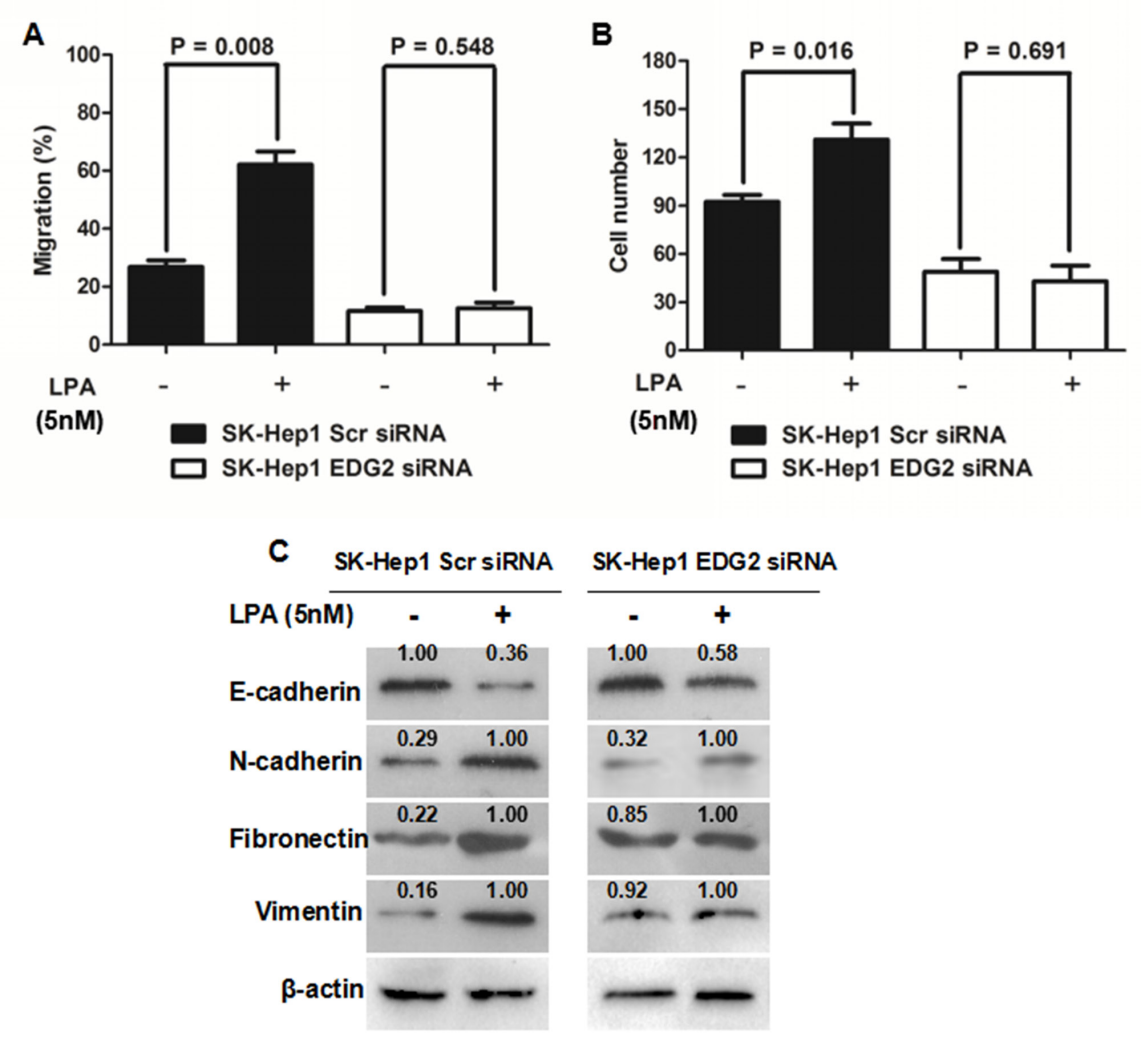
EDG2 played a vital role in LPA-induced EMT phenotype of SK-Hep1 cells **(A)** LPA treatment (5nM) resulted in increased migration ability of SK-Hep1 cells, while knockdown of EDG2 in SK-Hep1 cells abolished the impact of LPA on migration capacity of SK-Hep1. **(B)** Similiarly, invasion ability of SK-Hep1 cells was enhanced magnificently by LPA treatment, which was abated by knockdown of EDG2. **(C)** LPA decreased E-cadherin expression and increased the expression of N-cadherin, Fibronectin and Vimentin, while knockdown of EDG2 inhibited the influence of LPA on the expression of E-cadherin, N-cadherin, Fibronectin and Vimentin.

The reprogramming of choline catabolism and phospholipid metabolism during HCC pathogenesis resulted in up-regulation of LPA in HCC tissues [24]. On the basis of the findings mentioned above, it was reasonable to raise a hypothesis that LPA could induce EMT in the EDG2 (the LPA receptor)-dependent manner. As shown in Figure 4A, LPA treatment up-regulated TWIST, SNAI1, N-cadherin, Fibronectin and Vimentin and suppressed E-cadherin while increasing EDG2 expression. However, it did not affect the activation of TGFβ1 signaling. We treated SK-Hep1 cells with LPA (5 nM). As shown in Figure 4A and 4B, LPA treatment increased both cell migration and invasion capacities of SK-Hep1 Scr siRNA cells. Interestingly, LPA did not impact the migration and invasion abilities of SK-Hep1 EDG2 siRNA cells. LPA treatment also down-regulated E-cadherin expression in SK-Hep1 Scr siRNA cells while up-regulating the expression of N-cadherin, Fibronectin and Vimentin (Figure 4C). And no significant differences of the expression of these EMT markers were found between SK-Hep1 EDG2 siRNA cells with LPA or without LPA (Figure 4C). Thus, it demonstrated that LPA drove EMT phenotype of HCC cells via the EDG2 involved cell pathways.

### EDG2 induced EMT phenotype of HCC cells by activating PI3K/AKT/ mTOR pathway

**Figure 5:**
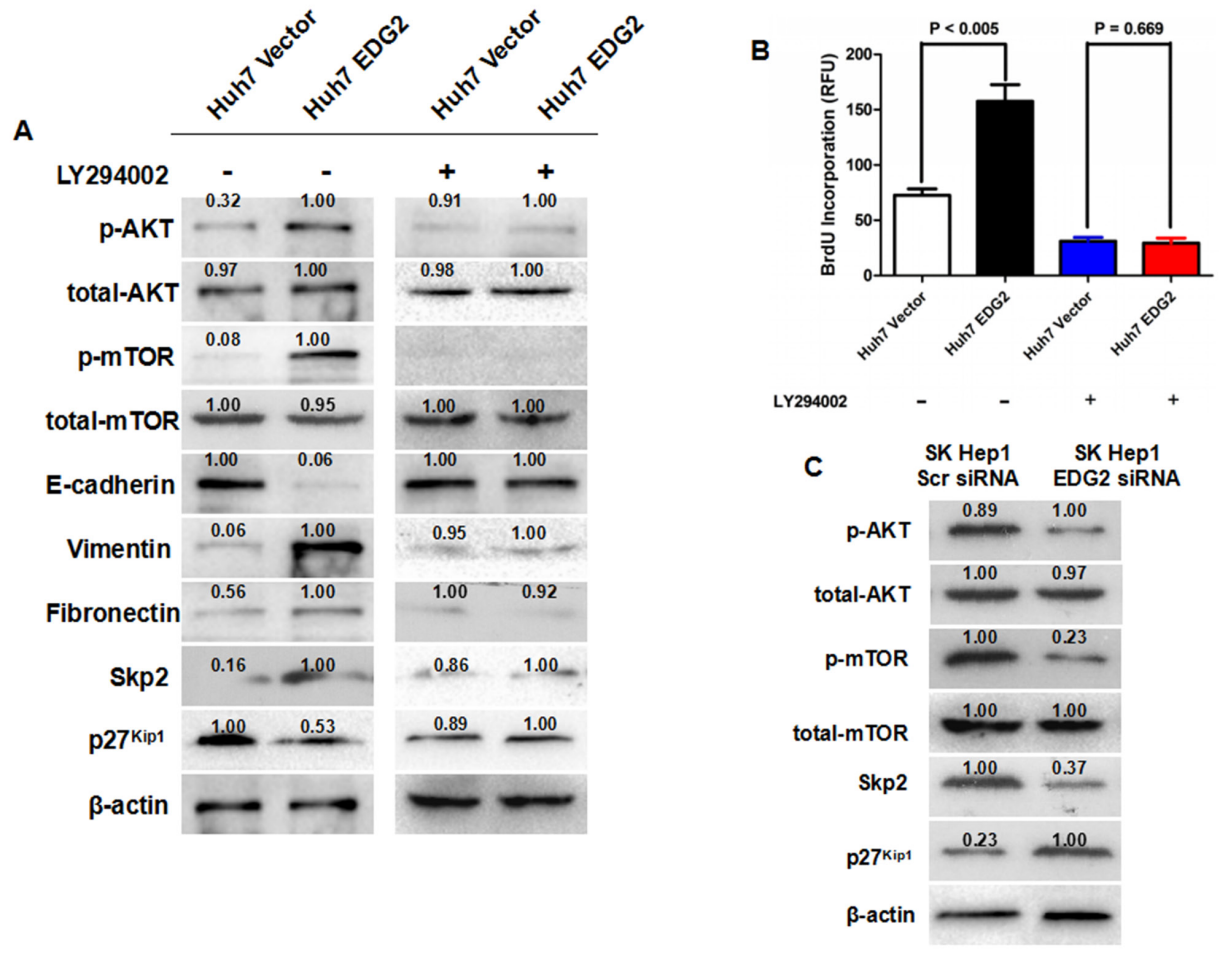
EDG2 mediated EMT and Skp2/p27^kip1^ axis through PI3K/AKT/mTOR signaling **(A)** As detected by Western immunoblotting, enhanced expression of EDG2 leaded to more phosphorylation of AKT and mTOR in Huh7 cells. Consequently, both decreased expression of E-cadherin and increased expression of Vimentin and Fibronectin was found after over-expression of EDG2 in Huh7. Skp2 expression was increased in Huh7 EDG2 cells, while p27^kip1^ expression was inhibited. However, the PI3K inhibitor LY294002 treatment abrogated the influence of EDG2 on the phosphorylation of mTOR, EMT markers and Skp2/p27kip1 axis. **(B)** BrdU Incoporation ELISA assay found that LY294002 treatment inhibited the proliferation of both Huh7 EDG2 and Huh7 Vector cells significantly. In addition, LY294002 abolished the pro-proliferation function of EDG2 on Huh7 cells. **(C)** Knockdown of EDG2 repressed the phosphorylation of both AKT and mTOR, decreased Skp2 expression and up-regulated p27(^kip1^.

The PI3K/AKT/mTOR axis was found to be activated by several intracellular signaling pathways, and consequently mediate several cellular events, including cell-cycle progression, proliferation/growth and EMT [25, 26]. Therefore, we investigated whether PI3K/AKT/mTOR pathway contributed into the oncogenic effect of EDG2 in HCC progression. Western immunoblotting assay showed that there was more p-AKT and p-mTOR detected in Huh7 EDG2 cells than Huh7 Vector cells (Figure 5A). And knockdown of EDG2 leaded to a remarkable decrease in the phosphorylation level of both AKT and mTOR in SK-Hep1 cells (Figure 5B). Furthermore, the PI3K inhibitor (LY294002) (10μM) inhibited the up-regulation of p-AKT and p-mTOR, and reverted the EMT phenotype induced by enforced expression of EDG2 in Huh7 cells (Figure 5A).

To confirm the regulatory function of EDG2 on activation of PI3K/AKT/mTOR signaling, we examined the change of Skp2, the downstream gene of PI3K/AKT/mTOR signaling [27–29]. Figure 5A showed that enhanced expression of EDG2 leaded to up-regulation of Skp2 expression in Huh7 cells. Knockdown of EDG2 also decreased the expression of Skp2 in SK-Hep1 cells (Figure 5B). The expression of p27^Kip1^, cyclin-dependent kinases (CDKs) inhibitor which was degraded by Skp2 via mediating its ubiquitylation [19, 30], was found decreased in Huh7 EDG2 cells compared with Huh7 Vector cells (Figure 5A). And LY294002 treatment abated the pro-proliferation of EDG2 on Huh7 cells (Figure 5B). Consistently, silencing EDG2 resulted in up-regulation of p27^Kip1^ expression in SK-Hep1 cells (Figure 5C). As expected, the effect of EDG2 on expression of both Skp2 and p27^Kip1^ in Huh7 cells was also eliminated by LY294002. These data supported that EDG2 promoted EMT and cell proliferation of HCC cells via activating PI3K/AKT/mTOR pathway.

### EDG2 enhanced tumor growth in HCC xenograft model

**Figure 6:**
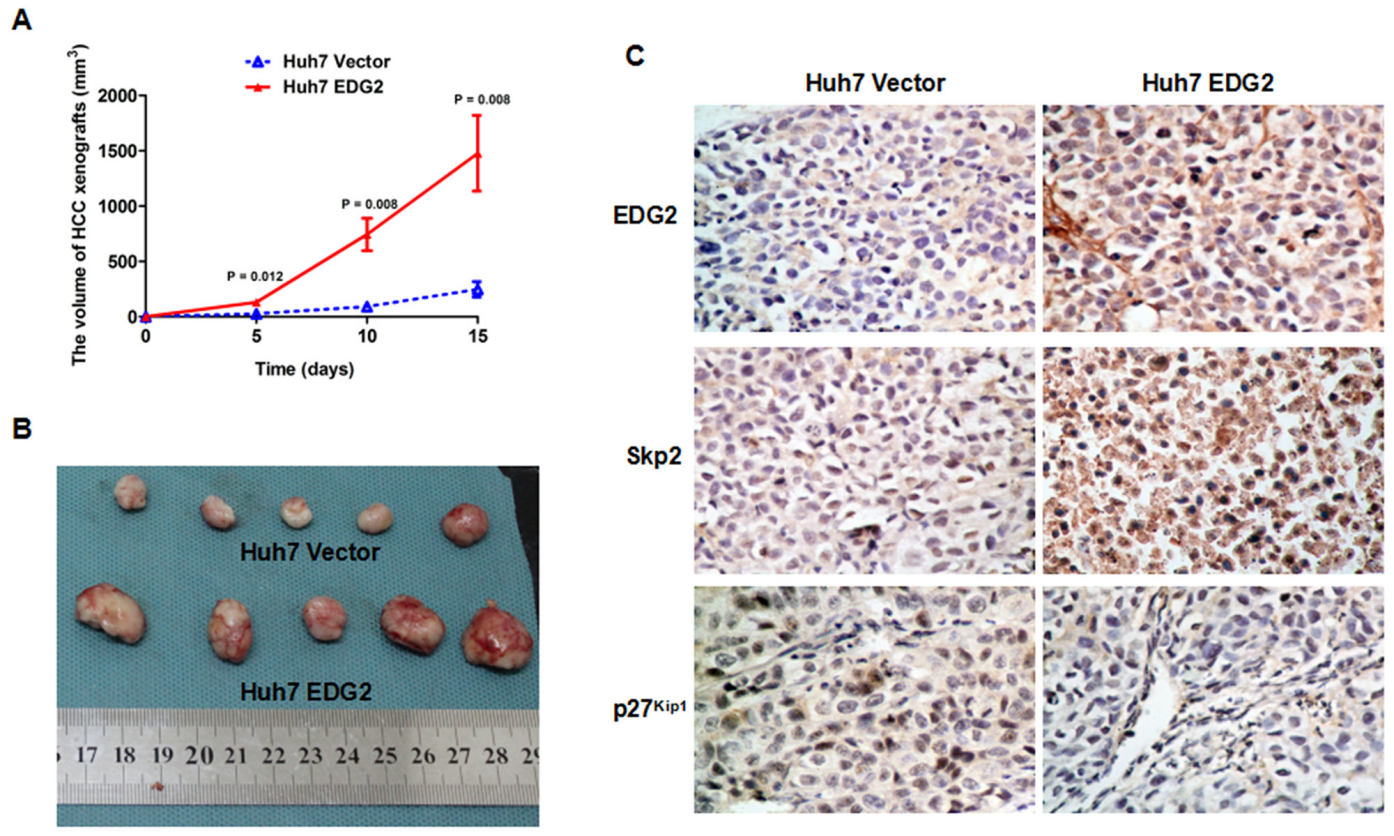
EDG2 over-expression accelerated HCC cell growth on nude mouse model **(A)** HCC xenografts driven from Huh7 EDG2 cells grew significantly faster that those from Huh7 Vector cells. **(B)** The size of HCC xenografts harvested from Huh7 EDG2 group were larger than those from Huh7 Vector group. **(C)** IHC staining assay displayed that there was more expressioin of EDG2 and Skp2 and less p27 kip1 expression in xenograft tissues from Huh7 EDG2 group compared with those from Huh7 Vector group (Amplification: ×400).

EDG2 was found to boost HCC cell proliferation via PI3K/AKT/mTOR pathway *in vitro* by the mentioned-above evidences in this study. To verify this effect of EDG2 on HCC progression *in vivo*, we built the HCC xenograft model with both Huh7 EDG2 cells and Huh7 Vector cells as mentioned in the Materials and methods section. As shown in Figure 6A, HCC xenografts from Huh7 EDG2 cells group (Huh7 EDG2 group) grew faster dramatically than those in Huh7 Vector cells group (Huh7 Vector group). On the 15^th^ day after HCC cell injection, the size of HCC xenografts driven from Huh7 EDG2 cell group was significantly larger than those from Huh7 Vector cell group, which was verified by Student’s t-test (Figure 6B). IHC staining assay on HCC xenograft tissues confirmed that more EDG2 was over-expressed in xenografts from Huh7 EDG2 group compared with Huh7 Vector group (Figure 6C). In the xenograft specimens obtained from Huh7 EDG2 group, there was more expression of Skp2 and lower level of p27^Kip1^ compared to Huh7 Vector group (Figure 6C). Therefore, these data demonstrated that EDG2 leaded to up-regulation of Skp2 and abolishment of p27^Kip1^, and consequently promoted HCC cell growth *in vivo*.

## DISCUSSION

Despite that a ton of important advances in the treatment of HCC have obtained during the last decades [31], liver resection remains the first-line curative treatment for HCC patients worldwide currently. However, prognosis after liver resection was still unsatisfied due to the high postsurgical rates of tumor recurrence and metastases [32]. Hence, it is urgent to explore the molecular mechanism of HCC recurrence and metastasis to identify the novel postsurgical predictive biomarkers and develop the new target therapy after liver resection. Several oncogenic factors for HCC play critical roles on liver development and regeneration, for instances SDF-1 [33], EGF [34], and NF-κB [35]. On the other hand, The recurrence of HCC has been considered to originate from either intrahepatic metastasis of primary tumor or de novo tumor arising from the remnant liver [36]. Therefore, it implied that the growth factors and/or chemokines up-regulated in liver remnants after liver resection could contribute to HCC recurrence after liver resection.

Simo et al. reported that serum LPA level was magnificently increased during 72h to 7 days after partial hepatectomy on the male C57BL/6 mouse model without any liver diseases. Moreover, after liver resection, EDG2 expression in the regenerating liver tissues was significantly higher than one in normal liver tissues in the sham-operation group [37]. These data demonstrated that LPA/EDG2 signaling was aberrantly activated in liver regeneration after liver resection. LPA signaling has been found to promote cancer progression in a variety of cancers including renal cancer [38], breast cancer [12], colon cancer [39] and ovarian cancer [40]. Similarly, Mazzoca et al found that LPA secreted by

HCC cells promoted HCC progression via recruiting peritumoral tissue fibroblasts and driving their transdifferentiation into myofibroblasts [41]. EDG2 was found over-expressed in HCC tissues compared with normal liver tissues and involved in promoting invasion capacity of HCC cell [11]. However, to the best of our knowledge, there have been no reports investigating the association between EDG2 expression and postsurgical outcome of HCC patients. In the present study, by the IHC staining assay, we also detected that there was significantly higher EDG2 expression in HCC tissues than adjacent liver tissues. Further analysis showed that HCC patients with higher EDG2 in tumor tissues had the lower 1-, 3-, and 5-year survival rates compared with those with less EDG2 in tumor tissues. And comparison of Kaplan-Meier survival curves displayed that over-expression of EDG2 in HCC tissues was correlated remarkably with the worse prognosis after liver resection.

The following *in vitro* studies showed that enforced expression of EDG2 accelerated HCC cell growth through promoting cell viability and proliferation, while siliencing EDG2 inhibited cell viability and proliferation of HCC cells. The related mechanistic investigation demonstrated that ectopic expression of EDG2 in Huh7 cells increased Skp2 expression and consequently repressed p27^Kip1^ expression via up-regulating phosphylation of AKT and mTOR. The PI3K inhibitor LY294002 suppressed the phosphylation of AKT and mTOR induced by ectopic expression of EDG2 and eliminated the impact of EDG2 over-expression on the expression of Skp2 and p27^Kip1^. The opposite results were obtained in the EDG2-knockdown experiments. The *in vivo* experiments also confirmed that enforced expression of EDG2 accelerated HCC xenograft growth and there was more Skp2 expression and less expression of p27^Kip1^ in xenograft tissues with the higher level of EDG2. These data demonstrated strongly that EDG2 promoted HCC cell viability and proliferation through activating PI3K/AKT/mTOR/Skp2/p27^Kip1^ signaling. p27^Kip1^ was a kind of cyclin-dependent kinases (CDKs) inhibitors and repression of p27^Kip1^/ CDK2 signaling has been found to contribute to hepatocarcinogenesis greatly by several evidences [42, 43]. The findings here also indicated that aberrant activated LPA/EDG2 signaling contributed to inhibition of p27^Kip1^/ CDK2 signaling.

There have been several studies revealing that LPA induced EMT phenotype of ovarian cancer cells via different mechanisms. Dhanasekaran group showed that LPA sensitized the Src/Gαi2 signaling pathway to result in up-regulation of HIF1α and finally initiated EMT phenotype of ovarian cancer cells [17]. Stack group found that LPA induced EMT in ovarian cancer cells through the β1-integrin-dependent Wnt/β-catenin signaling [18]. Although some studies clarified that LPA boosted the migration and invasion ability of HCC cells through the diverse molecular mechanisms [44, 45], no investigation has been reported about LPA and EMT of HCC cells yet. This study here revealed that forced expression of EDG2 promoted migration and invasion capacities of HCC cells and leaded to repression of E-cadherin and enhanced expression of N-cadherin Vimentin, and Fibronectin, while knockdown of EDG2 brought about the opposite results, which illustrated strongly that EDG2 elicited EMT of HCC cells. And LPA treatment also resulted in the notable EMT characteristics of HCC cells, however, LY294002 reversed the EMT induced by LPA with repression of AKT/mTOR signaling. These data suggested that LPA/EDG2axis played an important role in initiation and/or maintenance of EMT phenotype of HCC through PI3K/ AKT/mTOR signaling.

Taken together, the results in the present study showed that EDG2 was over-expressed aberrantly in HCC tissues, which predicted poor postsurgical outcome of HCC patients. Up-regulation of LPA and EDG2 during liver regeneration could accelerate the growth of metastased HCC cells in the remanent liver tissues via PI3K/AKT/mTOR/Skp2/ p27^Kip1^ signaling and reinforce the migration and invasion abilities of HCC cells via evoking EMT phenotype. These findings could provide a novel mechanism that EDG2 promoted HCC recurrence after liver resection, which would help to establish EDG2 as a predictive factor for postsurgical HCC recurrence and develop the innovative target therapy after liver resection.

## MATERIALS AND METHODS

### Clinical samples and follow-up data

A total of 210 patients who were diagnosed with HCC were recruited in this investigation. All HCC patients underwent liver resection for HCC by the same surgical team in Department of Hepatobiliary Surgery at the First Hospital of Xian Jiaotong University from January 2004 to June 2007. Written informed consent was obtained from all patients, and all protocols in this investigation were approved by the Xi’an Jiaotong University Ethics Committee according to the Helsinki Declaration of 1975.

Diagnosis of HCC was made according to the criteria of the Chinese Society of Liver Cancer and Chinese Anti-Cancer Association. No patients received any neo-adjuvant chemotherapy or radiotherapy before liver resection. Specimens of both HCC and adjacent liver tissues for the further assessments were obtained during liver resection and immediately stored at paraformaldehyde for immunohistochemistry staining assay. The demographic information from these 210 HCC patients were obtained from their medical records. The histopathologic information including Edmonson-Sterner classification, TNM grading, portal vein invasion, intra-hepatic metastases and tumor diameter was from two experienced pathologists who were blinded to the clinical information. We got the follow-up information from 175 of 210 HCCs (83.3%) with the median follow-up time was 42.5 months, ranging from 3 to 75.4 months. The demographic features and clinicopathological data are presented in Table 1.

### Immunohistochemistry

Immunohistochemistry (IHC) staining was carried out as described previously [19]. Briefly speaking, 4 μm-thick specimen slides for IHC staining were deparaffinized, and peroxidase was quenched with methanol and 3% H2O2 for 15 min. All sections were boiled with pressure in citrate buffer for 3 min and blocked overnight at 4°C. After incubated for 4 h with primary rabbit monoclonal anti-human EDG2 antibody (ab166903, Abcam, UK) at 1:100 dilution, the slides were washed with phosphate buffered saline (PBS), incubated with HRP-conjugated goat anti-rabbit antibody (ab6721; Abcam, UK) for 15 min at 1:100 dilution, and washed again with PBS. The staining of the slides was performed with the avidin-biotin-peroxidase complex. Finally, the sections were visualized with diaminobenzidine and counterstained with hematoxylin.

The scoring system for IHC staining was presented previously [16]. Staining intensity was divided into four grades: 0, none; 1, weak; 2, moderate; 3, strong. The percentage of specifically positive staining tumor cells was classified with the following grades: 0 (<5%), 1 (6%–25%), 2 (26%–50%), 3 (51%–75%), and 4 (>75%). The final score was expressed by multiplying the staining intensity and the percentage of specifically positive staining tumor cells.

### Western immunoblotting

Cell lysates were prepared in RIPA buffer containing inhibitors for proteases and sodium orthovanadate as an inhibitor of phosphatases. Western immunoblotting was conducted according to the protocol introduced previously [20]. The primary antibodies used in Western immunoblotting were listed in Supplementary Materials.

### Quantitative reverse-transcription-polymerase chain reaction (qRT-PCR)

cDNA synthesis and qRT-PCR examination were carried out as presented previously [21]. All samples were tested with an ABI 7300 system. GAPDH was simultaneously amplified in a separate set of tubes as the internal control. The primer sequences used in this study were as follows: EDG2: 5’-GCTGGTGATGGGACTTGGAA-3’ (forward) and 5’-GTCAATGAGGCCCTGACGAA-3’ (reverse); GAPDH: 5’-ACCACAGTCCATGCCATCAC-3′ (forward) and 5′-TCCACCACCCTGTTGCTGTA-3′ (reverse). Six replicates were taken in each assessment, and each was repeated three times.

### Cell culture

We obtained human HCC cell lines including SK-Hep1, Hep3B, HepG2 and PLC/PRF/5, and the human immortalized normal hepatocyte cell line LO2 from the Institute of Biochemistry and Cell Biology, Chinese Academy of Sciences (Shanghai, China) and Huh7 cells were a kind gift from Prof. Kefeng Dou (Department of Hepatobiliary Surgery, Xijing Hospital, Fourth Military Medical University). SK-Hep1, Hep3B, PLC/PRF/5 and LO2 were cultured in DMEM medium with 10% fetal calf serum (FBS) in a 5% CO2 humidified atmosphere at 37°C, while HepG2 was grown in RPMI 1640 medium with 10% FBS.

### Generation of HCC cells expressing EDG2

The EDG2 expressing plasmid was built by recombining the cDNA of human EDG2 into the pCMV-Tag2B vector (Stratagene, USA). EDG2 expressing plasmid was transfected into the wild-type Huh7 cells with low level of EDG2 (Huh7 EDG2 cells) with the FuGENE6 transfection reagent. Huh7 cells were transfected with the pCMV-Tag2B empty vector as Huh7 Vector cells. The stable transfection clones were selected with geneticin at a dose of 500 μg/mL for two weeks.

### Establishment of EDG2 knockdown cells

The Short interference RNA (siRNA) sequences against EDG2 (sc-43746) and the related scramble siRNA sequences (sc-37007) were both purchased from Santa Cruz Biotechnology. SK-Hep1 cells were plated at 5×10^4^ cells per 35-mm well for 24 h and respectively transfected with EDG2 siRNA (SK-Hep1 EDG2 siRNA) or scrambled siRNA (SK-Hep1 Scr siRNA) using the Lipofectamine RNA iMAX reagent (Invitrogen, USA). EDG2 knockdown was confirmed by both qRT-PCR and Western immunoblotting.

### Invasion assay and wound healing assay

Invasion ability of HCC cells was measured by transwell chamber invasion assay, while wound healing assay was conducted to measure cell migration. The details were described in the Supplementary Materials.

### Confocal microscopy and immunofluorescence

HCC cells were grown overnight and fixed with 4% paraformaldehyde for 30 min. PBS containing 0.2% Triton X-100 was use to permeabilize HCC cells for 20 min and 2.5% BSA buffer blocked cells for 2 h. HCC cells were incubated with primary antibodies to E-cadherin (ab40772, Abcam, 1:500 dilution) and Vimentin (ab45939, Abcam, 1:500 dilution) at 4°C overnight, and then stained with the different secondary antibodies including Alexa Fluor-488 or -647 (1:500 dilution, Invitrogen) for 4 h at room temperature. The nuclei of HCC cells was stained with DAPI. Immunofluorescent images were captured using a laser scanning confocal microscope (Carl Zeiss, Germany).

### BrdU incorporation and methyl thiazolyl tetrazolium assay

To detect the cell proliferation, BrdU incorporation assay was carried out with the BrdU ELISA kit from Abcam (Cambridge, MA, USA) according to the manual instructions [22]. The methyl thiazolyl tetrazolium (MTT) assay was conducted to measure the cell viability. HCC cells were grown at a concentration of 10^4^ per well in 96-well plates with 200 μl medium for 24, 48, 72 and 96 h. After removing culture medium, HCC cells were incubated with 0.25 mg/ml MTT in serum-free medium at 37°C for 4 h. The supernatant was removed and 200 μl of dimethyl sulfoxide was added to solubilize the formazan. The plates were shaken for 5 min and cell survival was assessed by measuring the value of OD570 with a colorimetric reader. The experiment with 6 replicates was conducted for three times independently.

### HCC xenograft experiments

In order to analyze tumorigenesis, HCC xenograft assay was performed on nude mouse model. A total of 10 nude mice with 6–8 weeks old were purchased from the Animal Experiment Center of Xian Jiaotong University (Xian, China) and divided into two groups randomly: Huh7 EDG2 group (n = 5) and Huh7 Vector group (n = 5). In the group of Huh7 EDG2, each mouse was injected subcutaneously with the cell mixture including 2×10^6^ Huh7 EDG2 cells and 150μL of Matrigel. Similarly, The cell mixture of 2×10^6^ Huh7 Vector cells and 150μL of Matrigel was injected subcutaneously into the flanks of nude mouse from Huh7 Vector group. Tumor sizes were measured with calipers every 5 days. Upon completion of the experiment (day 15), all mice from both groups were sacrificed. The volume of HCC xenografts was obtained using the following formula: volume = A × B^2^ × 0.52 (A, length; B, width; all measurements were in millimeters).

### Statistical analysis

All data were presented as means and standard errors of the mean. Differences between groups were compared with the Mann-Whitney test or Student-t test. Cox’s proportional hazards model was used to examine the prognostic value of EDG2 protein expression associated with overall survival. Univariable regression analyses were carried out with death as the outcomes. The Cox proportional hazards model with a stepwise procedure were used for multivariate analysis. Differences of the Kaplan-Meier curves between EDG2 high group and EDG2 low group were detected using the log rank test. Statistical significance was set at P < 0.05. Multivariate analysis was conducted with SPSS V17.0 software, and Graphpad PRISM 5 was used for other statistical analyses.

## SUPPLEMENTARY MATERIALS FIGURES


