# Supplementary material for: EDG2 enhanced the progression of hepatocellular carcinoma by LPA/PI3K/AKT/ mTOR signaling
Source: Oncotarget. 2017 Aug 2;8(39):66154–68. doi: 10.18632/oncotarget.19825 (PMC5630400; doi:10.18632/oncotarget.19825)
Supplement: Supplementary file 1 [file oncotarget-08-66154-s001.pdf]

EDG2 enhanced the progression of hepatocellular carcinoma by LPA/PI3K/AKT/ mTOR signaling

SUPPLEMENTARY MATERIALS

Antibodies for Western immunoblotting

The following primary antibodies were used in this investigation: rabbit monoclonal anti-human EDG2 antibody (ab166903, Abcam, 1:100 dilution), rabbit polyclonal anti-human p-AKT (T308) (ab38449, Abcam, 1:500 dilution), rabbit monoclonal anti-human p-AKT (S473) (ab81283, Abcam, 1:1000 dilution), rabbit monoclonal anti-human p-mTOR (S2448) (ab109268, Abcam, 1:1000 dilution), rabbit polyclonal anti-human AKT (ab64148, Abcam, 1:500 dilution), rabbit monoclonal anti-human mTOR (ab32028, Abcam, 1:2000 dilution), rabbit polyclonal anti-human Skp2 (ab68455, Abcam, 1:1000 dilution), rabbit monoclonal p27Kip1 (ab 32034, Abcam, 1:1000 dilution), anti-human rabbit monoclonal anti-human E-cadherin (ab40772, Abcam, 1:5000 dilution), rabbit polyclonal anti-human N-cadherin (ab18203, Abcam, 1:1000 dilution), mouse monoclonal anti-human Vimentin (ab45939, Abcam, 1:1000 dilution), rabbit polyclonal anti-human Fibronectin (ab2413, Abcam, 1:1000 dilution), and mouse monoclonal anti-humanβ-actin (ab8226, Abcam, 1:1000 dilution). β-actin was used to control for equal loading.

Invasion assay

The upper chamber of a 24-well transwell plate was pre-coated with Matrigel. Then, HCC cell suspension in serum-free medium was loaded into the top chamber at the concentration of 50,000 cells/mL, while the bottom chambers were filled with medium with 20% FBS. After 48 h incubation, HCC cells invaded the lower surface of the membrane were fixed with 4% paraformaldehyde, and stained with 0.05% Crystal Violet. The number of the migrated cells was counted in ten random distinct fields under a light microscopy.

Wound healing assay

HCC cells was seeded onto 24-well plates to approximately 80% confluence as a monolayer. The surface of the dishes was scratched linearly with a new 1000-μl pipette tip, and the gap distance was then measured by photographs. Images were captured with an inverted digital camera at 0 and 24 h. Migration capacity was examined by measuring the width of the wound at the same point.

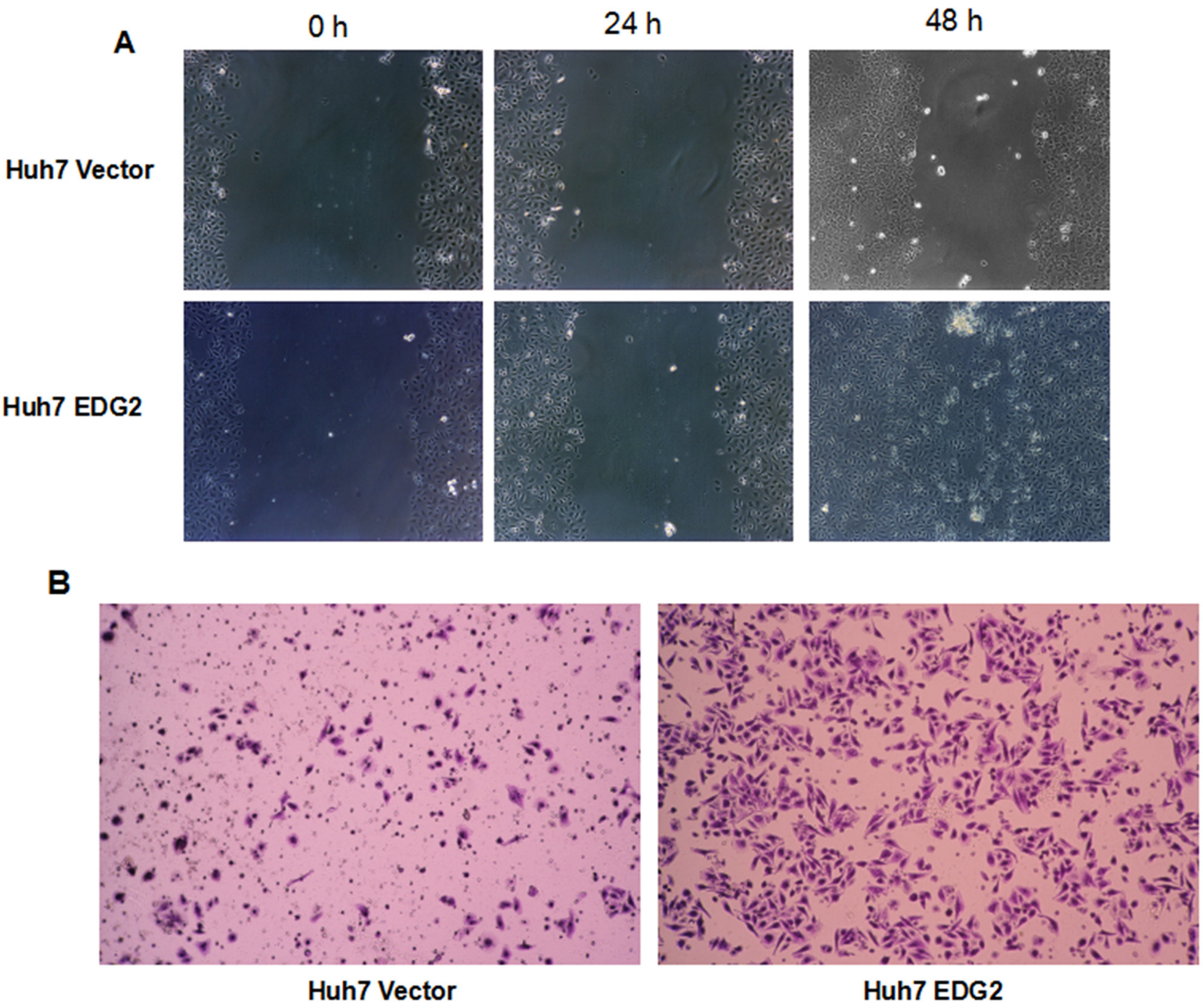

Supplementary Figure 1: The representative pictures of wound healing assay and Transwell invasion for Huh7 Vector and EDG2 cells. (A) Wound healing assay showed that the migration capacity of Huh7 cells was promoted by EDG2 over-expression. **(B)** Transwell assay displayed that Huh7 EDG2 cells possessed higher invasion ability than Huh7 Vector cells.

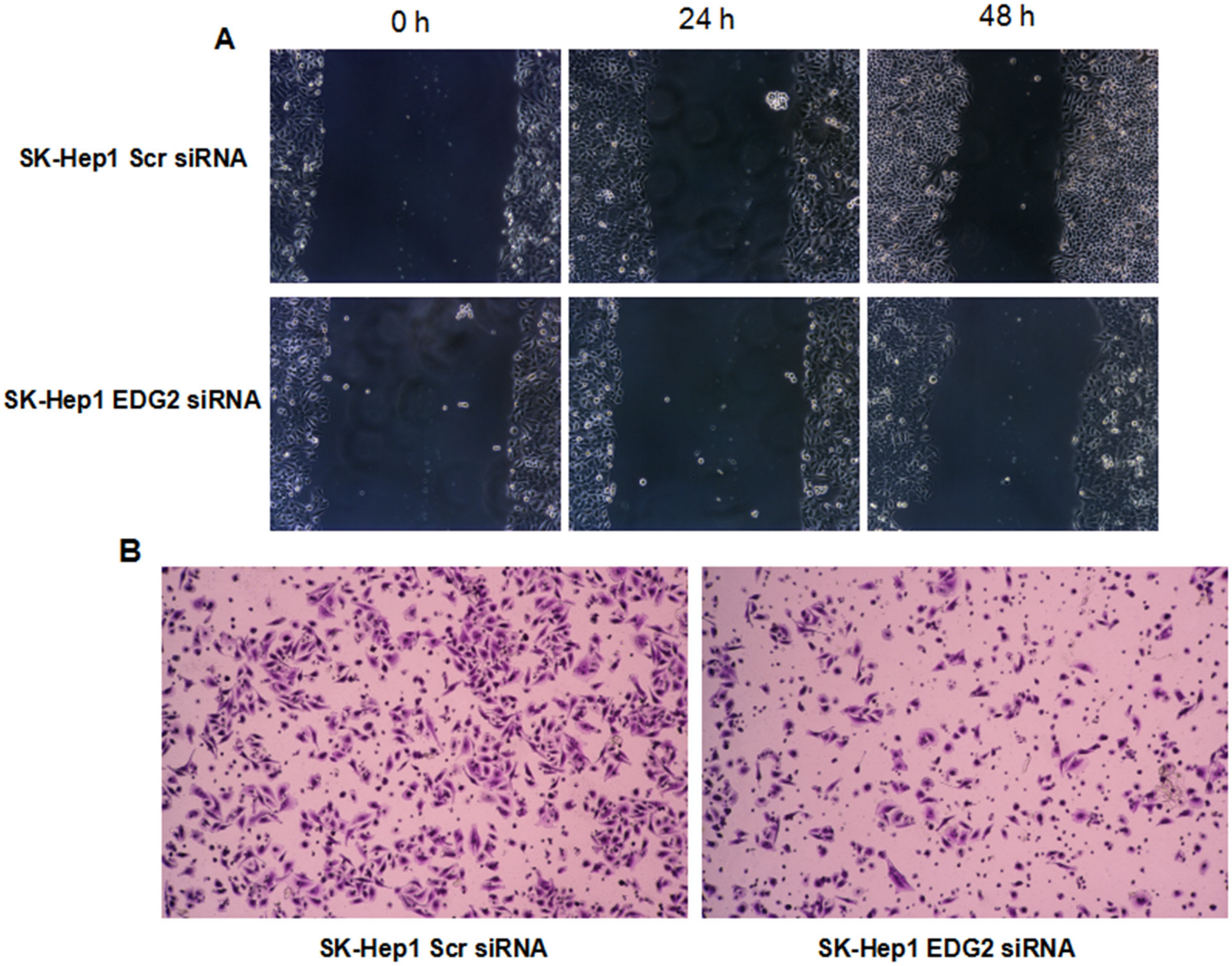

Supplementary Figure 2: The representative pictures of wound healing assay and Transwell invasion for SK-Hep1 Scr siRNA and SK-Hep1 EDG2 siRNA cells. **(A)** Wound healing assay showed that knockdown of EDG2 resulted in decrease of migration capacity of SK-Hep1 cells clearly. **(B)** Transwell assay displayed that silencing EDG2 repressed SK-Hep1 cell invasion notably.
